# Construction of S100 family members prognosis prediction model and analysis of immune microenvironment landscape at single-cell level in pancreatic adenocarcinoma: a tumor marker prognostic study

**DOI:** 10.1097/JS9.0000000000001293

**Published:** 2024-03-18

**Authors:** Zi-jin Xu, Jian-ang Li, Ze-yuan Cao, Hua-xiang Xu, Ying Ying, Zhi-hang Xu, Run-jie Liu, Yuquan Guo, Zi-xin Zhang, Wen-quan Wang, Liang Liu

**Affiliations:** a Department of Pancreatic Surgery, Zhongshan Hospital, Fudan University; b Cancer Center, Zhongshan Hospital, Fudan University; c Department of Oncology, Shanghai Medical College, Fudan University; d Department of General Surgery, QingPu Branch of Zhongshan Hospital, Fudan University; e State Key Laboratory of Genetic Engineering, Human Phenome Institute, Fudan University, Shanghai, People’s Republic of China

**Keywords:** gene family, nucleotide metabolism, pancreatic adenocarcinoma, tumor microenvironment

## Abstract

Pancreatic adenocarcinoma characterized by a mere 10% 5-year survival rate, poses a formidable challenge due to its specific anatomical location, making tumor tissue acquisition difficult. This limitation underscores the critical need for novel biomarkers to stratify this patient population. Accordingly, this study aimed to construct a prognosis prediction model centered on S100 family members. Leveraging six S100 genes and their corresponding coefficients, an S100 score was calculated to predict survival outcomes. The present study provided comprehensive internal and external validation along with power evaluation results, substantiating the efficacy of the proposed model. Additionally, the study explored the S100-driven potential mechanisms underlying malignant progression. By comparing immune cell infiltration proportions in distinct patient groups with varying prognoses, the research identified differences driven by S100 expression. Furthermore, the analysis explored significant ligand-receptor pairs between malignant cells and immune cells influenced by S100 genes, uncovering crucial insights. Notably, the study identified a novel biomarker capable of predicting the sensitivity of neoadjuvant chemotherapy, offering promising avenues for further research and clinical application.

## Introduction

Highlights

- Family genes predicts the curative effect of most malignant tumor.
- The new therapy target of pancreatic cancer.
- The new marker of neoadjuvant therapy.

Pancreatic adenocarcinoma, a highly malignant neoplasm, exhibits a 5-year survival rate of ~10%^1^ and is steadily becoming the third leading of cause of cancer related mortality^2^. Surgical resection remains the mainstay of treatment for curative therapy^3^. In the realm of multidisciplinary treatment beyond surgery, adjuvant chemotherapy, employing agents such as gemcitabine, fluorouracil, and calcium leucovorin, is applied for resectable pancreatic adenocarcinoma^4^. For borderline resectable cases, neoadjuvant chemotherapy, utilizing FOLFIRINOX and the AG regimen (albumin paclitaxel combined with gemcitabine), is considered^5^. Despite the conventional TNM staging system provided by AJCC/UICC, which forms the basis of cancer progression classification, patients with the same stage exhibit diverse clinical features and outcomes^6^. The immune system’s pivotal role in the tumor microenvironment of pancreatic adenocarcinoma is widely acknowledged^7^. While initially identified as an immunologically cold tumor^8^, pancreatic adenocarcinoma can be categorized into immunologically active subsets using immune-related signatures^9^. Recent advancements in genome-sequencing technology have led to successful clinical applications of immune checkpoint inhibitors^10^. Indeed, the anatomical site of the pancreas contributes to challenges in tumor tissue acquisition, marked by difficulty and poor specificity^11^. Conventional serum biomarkers CEA and CA 19-9 offer limited diagnostic efficacy for malignancy degree and predictive accuracy for chemotherapy response^12–14^. Consequently, there is an urgent need for novel detection approaches to stratify patients with pancreatic adenocarcinoma and identify biomarkers predicting chemotherapy response.

The S100 family, comprising over 20 members, encoded by separate genes^15^, consists of small, dimeric, EF-hand type proteins with Ca^2+^ binding domains. These proteins play diverse roles in intracellular and extracellular functions, including calcium balance, proliferation, migration, apoptosis, differentiation, energy metabolism, protein phosphorylation, and inflammation^16^. Most S100 genes (*S100A1-S100A16*) reside in chromosome locus 1q21, while four others (S100B, S100G, S100P, and S100Z) are located in chromosome loci 21q22, Xp22, 4p16, and 5q14, respectively^17^. S100 family members have been associated with prognosis and play crucial roles in the progression of various tumors, making them potential tumor markers for stratifying patients with pancreatic adenocarcinoma^18^.

Because of the hardship in diagnostic sample acquisition and prognosis judgment, clinical diagnosis were badly in need of accurate and reliable biomarker for the construction of stratification system. The system would make sample acquisition and biomarker detection applied for individualized therapies. To date, there is no unified prediction model explaining the weights of S100 family members in prognosis prediction. Additionally, a comprehensive correlation analysis between S100 family members and the immune microenvironment remains lacking. Based on previous studies of S100 family members prognostic significance and potential mechanism exploration, we would provide an united prediction model with members emphasis from analyzing S100 family whole members prognosis. The prediction model could provide stratification system of patients with comprehensive therapy of pancreatic adenocarcinoma.

## Materials and methods

### Publicly available sample data

Data for this study were sourced from the Gene Expression Ominibus (https://www.ncbi.nlm.nih.gov/geo/), which provided microarray data and sample information from various cohorts, including datasets GSE71729^19^, GSE57495^20^, GSE79668^21^, GSE62452^22^, and GSE202051^23^.

In GSE71729, RNA expression matrices from 145 primary and 61 metastatic pancreatic ductal adenocarcinoma samples, 17 pancreatic cancer cell lines, 46 normal pancreatic tissue samples, and 88 distant site adjacent normal samples were included along with follow-up information until 2015. Chen *et al*. extracted RNA from 63 pancreatic ductal adenocarcinoma samples dataset GSE57495 for microarray analysis to identify a prognostic signature. Kirby MK analyzed the transcriptome of 51 human pancreatic adenocarcinoma cancer tissues samples in dataset GSE79668, aiming to reveal key genes associated with novel expression patterns linked to long-term survival. Yang *et al*. provided the gene expression profile of 65 pancreatic cancer samples with explicit follow-up information, focusing on the MIF signaling pathway’s role in driving the malignant character of pancreatic cancer in GSE62452. Massachusetts General Hospital published single-nucleus RNA-seq data in dataset GSE202051, including 18 specimens that received no treatment prior to resection and 25 specimens that underwent neoadjuvant chemoradiation therapy before resection.

Additionally, International Cancer Genome Consortium (ICGC) contributed PACA-AU (*n*=266) and PACA-CA (*n*=186) cohorts, providing RNA sequencing matrices and clinical information. The Cancer Genome Atlas (TCGA) dataset, encompassing over 20 000 primary cancers and matched normal samples across 33 cancer types, included 186 patients diagnosed with pancreatic adenocarcinoma between 2001 and 2013. From this dataset, 168 tissue samples with resection or biopsy and follow-up information were collected (https://www.cancer.gov/ccg/research/genome-sequencing/tcga). If the analysis incorporated the AJCC TNM stage system, only 165 samples contained the whole relevant information. The total of samples in Cox regression model was 165.

### Least Absolute Shrinkage and Selection Operator (LASSO) Cox regression

We employed the LASSO Cox regression, a classical method, to develop a prognosis prediction model^24^. This regression analysis technique incorporates both variable selection and regularization, aiming to improve the prediction accuracy and interpretability of the resulting statistical model. The model can establishes connections to ridge regression and soft thresholding, elucidating the relationships between LASSO coefficient estimates and the concept of soft thresholding.

### Receiver Operating Characteristic (ROC) curve and Area Under the Curve (AUC)

An ROC curve is visually represents the performance of a classification model across various classification thresholds. It plots included two parameters: the true positive rate and false positive rate. The AUC quantifies the overall two-dimensional area beneath the entire ROC curve, ranging from (0,0) to (1,1)^25^. In our study, we constructed time-dependent ROC curves for the S100 model, comparing AUC between S100 scores and single-gene prognosis. Additionally, we generated ROC curves to compare the efficacy of neoadjuvant therapy response between S100 subgroups and the AJCC stage system.

### Calibration curve

The calibration curve is a scatter plot of true positive and predicted positive values, providing a visual representation of the results of Hosmer–Lemeshow goodness-of-fit test to evaluate logistic regression and Cox regression models^26^. Our study included a calibration comparison between S100 scores and single-gene survival predictions.

### Bootstrap resampling

Bootstrap resampling involves random sampling with replacement, and falling under the broader category of resampling methods. This technique assigns accuracy measures to sample estimates, allowing the estimation of the sampling distribution of various statistics through random sampling. We validated the internal sub-sample of the S100 model and provided R^2^ values.

### Expression of cell clusters in single-cell levels analysis procedure

Utilizing the R package Seurat, we conducted quality control, analysis, and exploration of single-cell RNA sequencing data. Seurat facilitates the identification and interpretation of sources of heterogeneity from single-cell transcriptomic measurements, enabling the integration of diverse types of single-cell data^27^. Our analysis focused on the gene expression quantities at the single-cell level within the GSE202051 cohort.

### Calculation of feature scores at single-cell levels

We employed the AddModuleScore function to calculate the average expression levels of each cluster at the single-cell level, inputting gene sets to derive scores for each cell. This process allowed us to classify malignant cells into positive(+) and negative(-) cells based on score features.

### Differentiation expression analysis

The limma package, a data analysis linear model was utilized to analyze differential expression genes between the high and low S100 score groups^28^.

### Gene set enrichment analysis (GSEA) function

GSEA is a computational method assessing whether a predefined set of genes exhibits a statistically significant, and concordant difference between two biological states (e.g. high and low S100 score groups)^29^.

### Cell-cell interaction network analysis

The CellPhoneDB is a resource for inferring cell-cell communication based on the expression combination of multisubunit ligand-receptor pairs^30^. To analyze intercellular communication, we utilized the R package CellChat, grafting the CellPhoneDB database between different cell types^31^. Our focus centered on both direct and indirect interactions between malignant cells and immune cells, specifically CD8^+^ T cells, CD4^+^ T cells, and NK cells.

### Cells and reagents

PDAC cell lines (Capan-1) were obtained from the American Type Culture Collection (ATCC). Capan-1 cells were cultured in Iscove’s Modified Eagle’s Medium (IMEME) with 20% fetal bovine serum (Gibco, Thermo Fisher Scientific) and 1% penicillin-streptomycin.

Cells were treated with increasing of gemcitabine (ranging from 0.1 μM to 12.8 μM) for 24 h. The cell viability was detected by cell counting Kit-8 (CCK-8) reagent. With the analysis of cell viability data after gemcitabine treatment in pancreatic cells, the half maximal inhibitory concentration (IC50) was 0.82 μM.

Cells were treated with increasing of 5-fluorouracil (5-FU) (ranging from 1 μM to 128 μM) for 48 h. The half maximal inhibitory was calculated as 3.41 μM with the above mentioned method.

### Generation of gemcitabine-resistant cell lines

We generated the gemcitabine-resistant cell lines from the parental Capan-1 pancreatic cancer line by incrementally increasing the gemcitabine concentration in the culture medium over extended periods of time. The half maximal inhibitory was calculated as 1.55 μM with the above mentioned method.

### RNA sequencing

Capan-1 cells were treated for 24 h with 3.66 μM gemcitabine and for 48 h with 3.41 μM 5-FU. Total RNA was extracted using TRIZOL reagent (Invitrogen). Ribosomal RNA (rRNA) was removed with the Ribo-Zero Magnetic kit for RNA-Seq from EpiCentre. TruSeq RNA Sample Preparation was performed on the RiboMinus RNA fraction. The libraries were sequenced on an Illumina Novaseq 6000 instrument. The quality of RNA-seq libraries was assessed using fastQC. Reads were aligned to the human genome release version hg38 reference genome using STAR with default settings and annotated using the Ensembl (release-105) annotation.

### Remark criteria statement

Our research adheres to the Reporting Recommendations for Tumor Marker Prognostic Studies (REMARK) criteria (Supplemental Digital Content 1, http://links.lww.com/JS9/C172), ensuring transparency and comprehensive reporting of our study findings^32^.

## Results

### Construction of S100 family members prognosis prediction model and comparison of prediction efficacy

**Figure 1:**
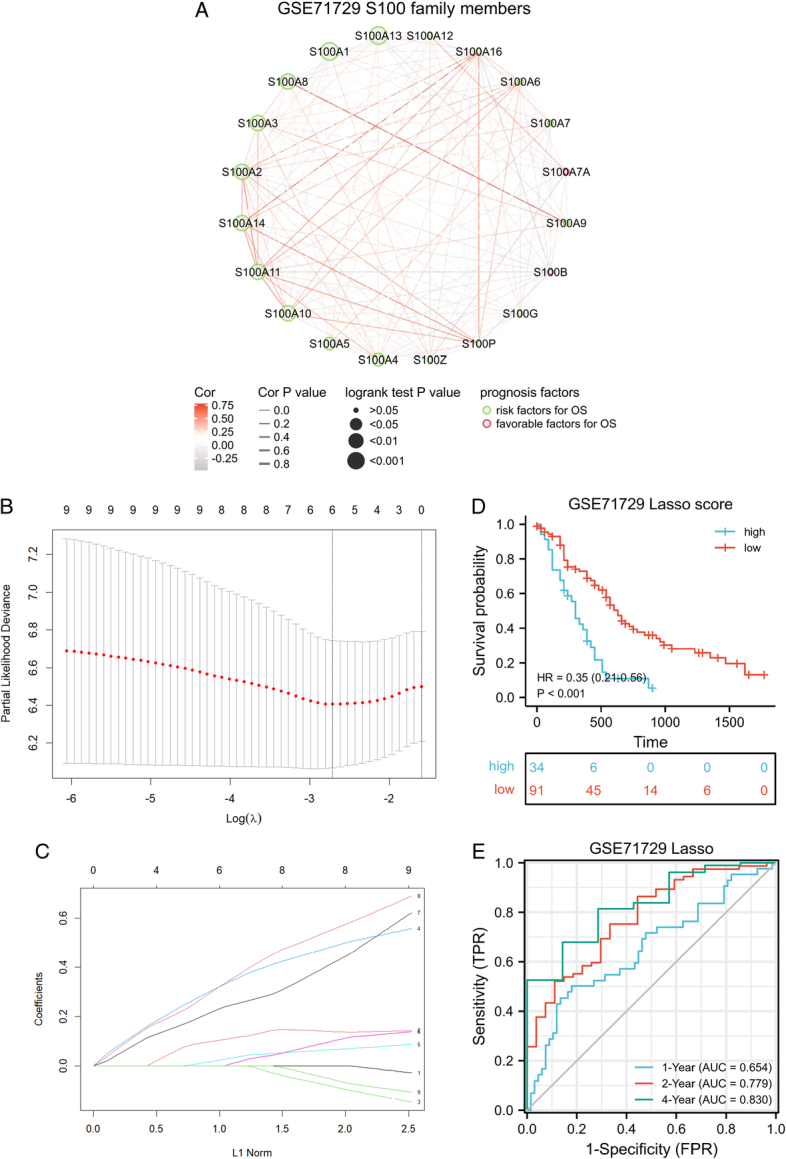
Construction of the interaction network and prognosis model of S100 family members in pancreatic adenocarcinoma. (A) The interaction network of S100 family members. The color of the edge denotes the Cor value. The width of the edge denotes the *P*-value. The shape of the node denotes S100 family members. The stroke color of the node denotes the favorable or risk factors for OS. The size of the node denotes the log-rank test *P*-value for prognosis curves. (B) LASSO regression with 10-fold cross-verification. (C) LASSO coefficient profiles of S100 family members. (D) The OS curve between high and low S100 score group in GSE71729 cohort. (E) The ROC curves of S100 score in 1, 2, and 4-year.

Initially, we constructed a network diagram (Fig. 1A) to visualize the interactions among S100 family members and their prognosis significance in the GSE71729 cohort. This analysis revealed 10 S100 family members with poor prognosis significance (Figure S1, Supplemental Digital Content 2, http://links.lww.com/JS9/C173). Subsequently, we assessed the prognosis significance of these 10 S100 family members in various cohorts, including TCGA, E-MTAB-6134, GSE57495, GSE79668, GSE62452, and ICGC. Notably, S100A1 did not emerge as a significant risk prognosis factor in these additional cohorts (Table 1). To identify key genes for our prognosis prediction model, we utilized the LASSO function in the R package glmnet. This approach facilitated the screening of hub genes and the certification of coefficients. We presented the LASSO regression results with 10-fold cross-verification and the corresponding coefficient profiles for S100 family members (Fig. 1B–C). Subsequently, the finalized prediction model comprised 6 S100 family members, with each gene associated with a specific risk coefficient (Table 2). We calculated the S100 score for each sample, revealing that the subgroup with a high S100 score exhibited a poor prognosis (Fig. 1D). To assess the predictive performance of our model, we generated ROC curves for the S100 score and calculated AUC values for 1, 2, and 4 years of survival (Fig. 1E). Importantly, we confirmed the prognostic significance of the S100 score in other cohorts (Figure S2A-F, Supplemental Digital Content 3, http://links.lww.com/JS9/C174). Additionally, we conducted univariate and multivariate Cox regression analyses for TCGA and GSE79668 cohorts, incorporating sex, age, and TNM stage information. Our analyses validated the subgroup stratification based on the S100 score as an independent prognostic factor (Figure S2 G–H, Supplemental Digital Content 3, http://links.lww.com/JS9/C174).

**Table 1:** External validation of prognosis significance of 10 S100 family members in other cohorts.

S100 family	Prognosis	*P*	Validation cohorts
S100A1	Risk	<0.001	NA
S100A2	Risk	0.001	TCGA/E-MTAB-6134/GSE57495/GSE79668/ICGC-AU/ICGC-CA
S100A3	Risk	0.008	GSE79668/ICGC-AU
S100A4	Risk	0.038	E-MTAB-6134/ICGC-AU/ICGC-CA
S100A5	Risk	0.017	GSE57495
S100A8	Risk	0.008	E-MTAB-6134/ICGC-AU/ICGC-CA
S100A10	Risk	0.001	GSE57495/GSE79668/ICGC-AU/ICGC-CA
S100A11	Risk	0.004	TCGA/GSE62452/GSE79668/ICGC-AU/ICGC-CA
S100A13	Risk	<0.001	GSE57495
S100A14	Risk	0.005	GSE57495/GSE62452/GSE79668

**Table 2:** S100 score calculation based on LASSO model.

S100 family members	Coefficient
S100A3	0.107054
S100A5	0.330630448
S100A8	0.025828887
S100A10	0.000476784
S100A11	0.239327356
S100A13	0.333980454

The S100 protein family members have been identified as significant contributors to the progression of pancreatic adenocarcinoma^33^. In our study, we integrated the predictive efficacy of S100 family members by constructing a comprehensive prognosis model. Next, we sought to explore the mechanisms underlying the malignant progression driven by S100 genes in pancreatic adenocarcinoma.

### Evaluation of prediction power and exploration of clinical therapy significance

**Figure 2:**
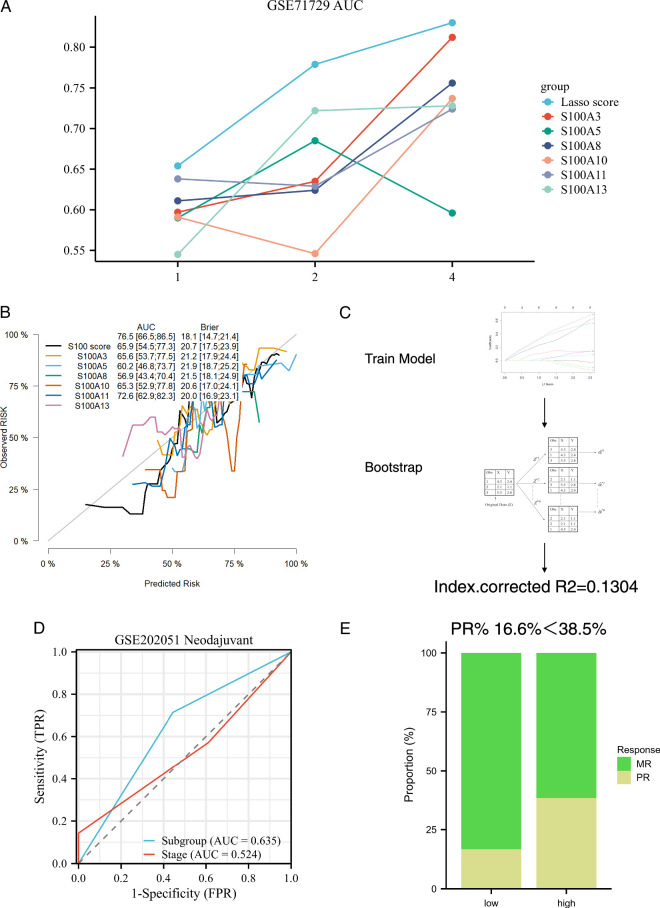
Power evaluation and clinical significance validation of the S100 score model. (A) The survival prediction efficacy with AUC comparison between S100 score and single gene. (B) Comparison of calibration curves between the S100 score and single gene predictions for survival outcomes. (C) Procedure and R2 calculation results of bootstrap-resampling validation for the S100 score model. (D) Prediction efficacy of poor response to neoadjuvant therapy, with AUC comparison between the S100 subgroup and AJCC Stage system. (E) Proportion comparison of poor response between S100 subgroups.

To assess the predictive power of our model and explore its clinical therapy significance, we employed several comprehensive analyses. Initially, we calculate the AUC values in 1, 2, and 4 years of survival using the time-dependent ROC curve, for single gene prognosis. By comparing these values, we illustrated the superior discrimination of our model (Fig. 2A). Subsequently, we generated a calibration curve for the S100 score and single gene prognosis prediction. The results indicated enhanced calibration, characterized by higher AUC values and lower Brier values compared to single gene predictions (Fig. 2B). Internal validation conducted through Bootstrap resampling in the prediction model of the training cohort, further confirmed the robustness of our model (Fig. 2C). A comparison of the correlation index (R^2^) between the S100 score and single genes validated a higher correlation with the survival outcome for the S100 score (Table S1, Supplemental Digital Content 4, http://links.lww.com/JS9/C175). To evaluate the predictive accuracy of 0.5-year and 1-year survival in the train cohort GSE71729, we calculated the true positive (TP), false positive (FP), false negative (FN), and true negative (TN) values for calculating positive predictive value (PPV), and negative predictive value (NPV) (Table 3), TCGA and GSE79668 cohorts demonstrated a higher PPV and NPV than AJCC Stage System for predicting 0.5-year and 1-year survival outcome (Table S2–S3, Supplemental Digital Content 5, http://links.lww.com/JS9/C176, Supplemental Digital Content 6, http://links.lww.com/JS9/C177). Because of independent prognostic factors including age, N stage, and S100 subgroup in TCGA cohort Cox regression model, we construct the nomogram to integrate the factors and optimize the model (Figure S3A, Supplemental Digital Content 7, http://links.lww.com/JS9/C178). Our study validated the RiskScore from nomogram with better discrimination and calibration than only S100 score prediction (Figure S3 B–D, Supplemental Digital Content 7, http://links.lww.com/JS9/C178). We also provided precision indexes of RiskScore in TCGA cohort (Table S4, Supplemental Digital Content 8, http://links.lww.com/JS9/C179). Furthermore, in terms of clinical therapy significance, our prediction model exhibited superior discrimination compared to the AJCC TNM stage for poor response to neoadjuvant therapy (Figure 2 D–E).

**Table 3:** The index of S100 model accuracy degree in the GSE71729 cohort.

	True	
0.5-year survival prediction	Positive	Negative
Prediction		
Positive	70	21
Negative	25	9
1-year survival prediction	True	
	Positive	Negative
Prediction		
Positive	55	36
Negative	13	21

0.5-year survival prediction.

ACC=0.632.

PPV=0.769

Sensitivity=0.737.

Specificity=0.3.

NPV=0.265.

1-year survival prediction.

ACC=0.608.

PPV=0.604.

Sensitivity=0.809.

Specificity=0.368.

NPV=0.618.

Our validation and comparison results of the S100 score model emphasize its significance in predicting both survival outcomes and neoadjuvant therapy responses. The efficacy of our model can be attributed to the oncogenic nature of hub S100 genes, underscoring the necessity to explore the malignant potential mechanisms of S100 genes.

### Distribution of S100 genes expression in cell clusters and exploration of S100-driven malignant tendency in single-cell levels

**Figure 3:**
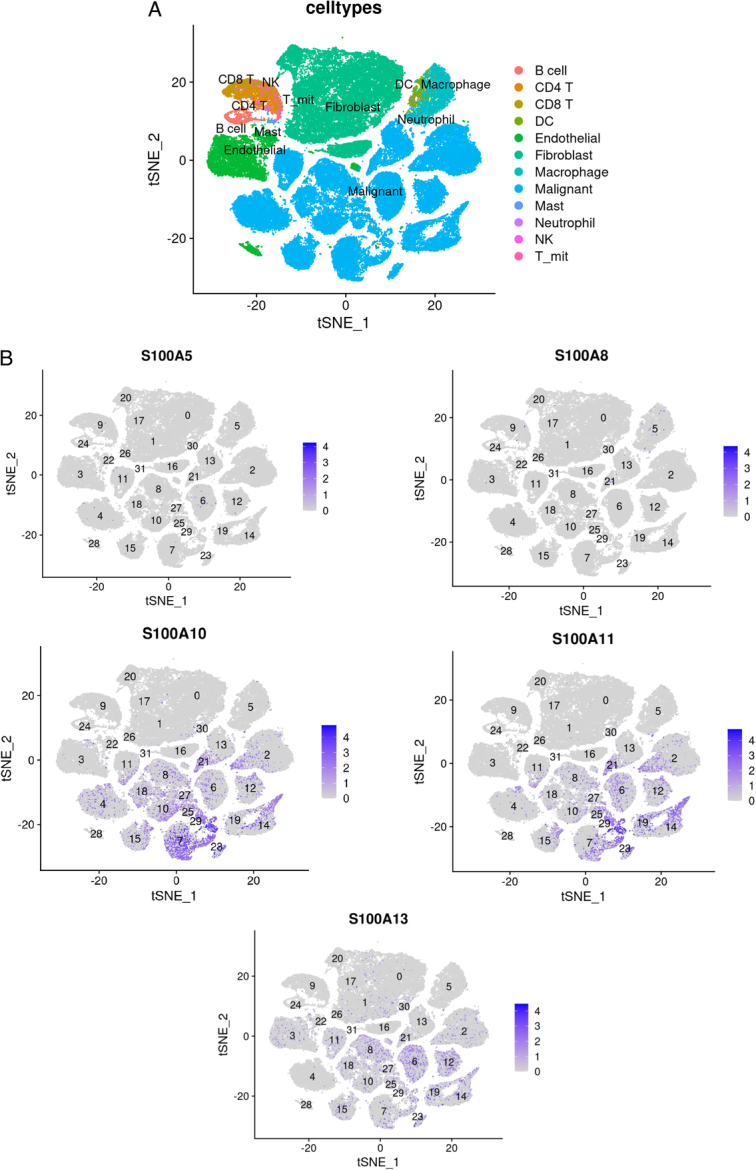
The single-cell transcriptomics expression analysis of S100 family members. (A) Distribution of cell clusters in the untreated group of GSE202051 cohort. tSNE embedding of single-cell dots of PDAC tumors colored by cell type. (B) The expression levels of S100 family members.

**Figure 4:**
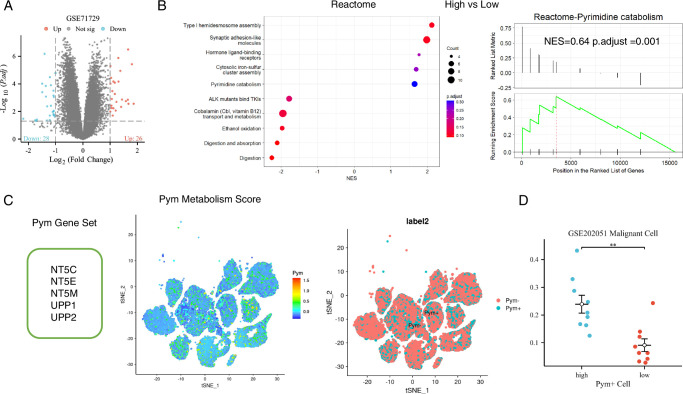
Potential mechanisms of the impact of the S100s score on malignant cells in pancreatic adenocarcinoma. (A) The volcano plot of differentially expressed genes in malignant cells between high and low S100 scores groups. (B) The dot plot of upregulation and downregulation top5 pathways from Reactome enrichment analysis results of malignant cells between high and low S100 scores group. (C) The calculation of pyrimidine metabolism in malignant cells. (D) The comparison of Pym+ malignant cells between high and low S100 score groups.

We used the R package Seurat to display mesenchymal microenvironment cells distribution at single-cell levels (Fig. 3A). The hub genes expression levels in cell clusters displayed in Figure 3B. To identify the genes associated with the S100-driven malignant tendency, we utilized the R package limma to analyze differential expression between high and low S100 score groups, resulting in the identification of 26 upregulated genes and 28 downregulated genes (Fig. 4A). Subsequently, Reactome enrichment analysis using GSEA revealed that the pyrimidine catabolism pathway could be a core mechanism underlying the S100-driven malignant tendency (Fig. 4B). Notably, pyrimidine nucleotides are integral to DNA synthesis and cell proliferation^34^. It has been reported that pyrimidine antimetabolites such as gemcitabine, capecitabine, and 5-FU are employed in adjuvant chemotherapy for pancreatic adenocarcinoma^35,36^. The observation that S100 genes may drive pyrimidine metabolism, consequently promoting the malignant potential in pancreatic adenocarcinoma, led us to identify seven pyrimidine catabolism-related enrichment genes. By displaying the gene expression levels in cell clusters, we identified five genes NT5C, NT5E, NT5M, UPP1, and UPP2 based on their expression levels in malignant cells. This set of genes was harnessed to classify pyrimidine metabolism-related cell subtypes (Figure S4, Supplemental Digital Content 9, http://links.lww.com/JS9/C180). Employing the AddModuleScore function in the R package Seurat, we calculated the pyrimidine metabolism score based on this gene set. Subsequently, the sctyper function was applied to classify malignant cells into Pym^+^ and Pym^-^ cells (Fig. 4C). Intriguingly, our findings revealed a discovered higher proportion of Pym+ malignant cells in high S100 score group (Fig. 4D), validating our initial hypothesis. Given the recognized significance of the tumor microenvironment in both experimental and clinical research of pancreatic adenocarcinoma^7^, it becomes imperative to explore the changes in the tumor immune microenvironment driven by S100 gene family members integrated into the prognosis model. S100 family members, known for their roles as alarmins, antimicrobial peptides, proinflammation stimulators, chemo-attractants, and metal scavengers^37^, are likely to exert a substantial influence on the tumor immune microenvironment. Therefore, a comprehensive exploration of these changes is essential for a comprehensive understanding of the implications of S100 gens in the context of pancreatic adenocarcinoma prognosis.

### Comprehensive analysis of S100-associated immune microenvironment change and CellChat analysis between key immune cells in pancreatic adenocarcinoma

**Figure 5:**
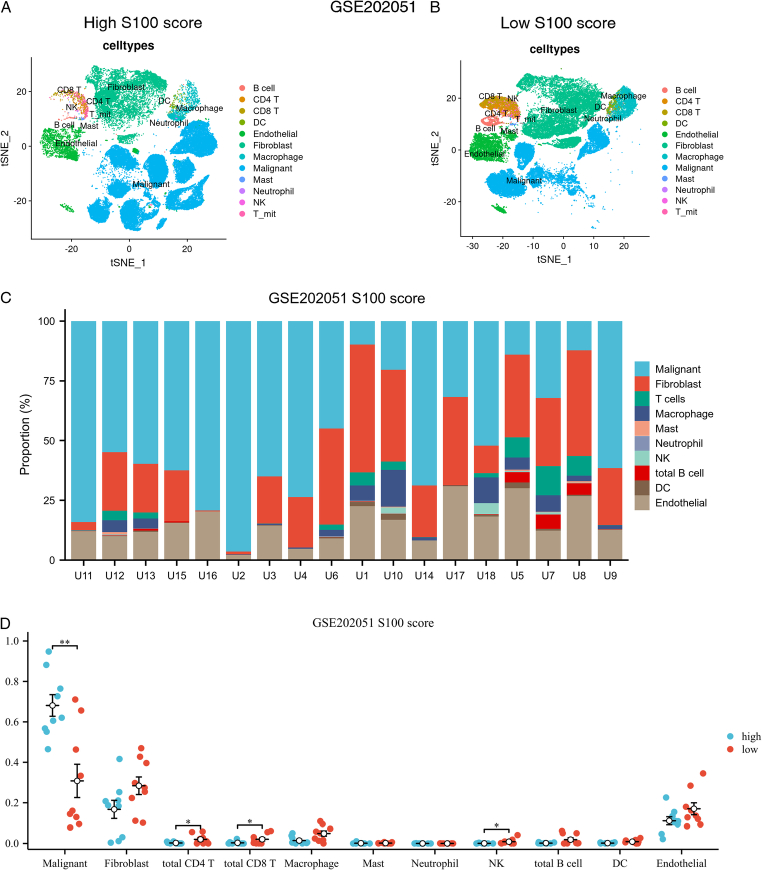
The immune microenvironment difference based on single-cell transcriptomic levels between high and low S100 score groups. (A–C) The cell clusters distribution between high and low S100 score groups. (D) The cell proportions in each tumor sample between high and low S100 score groups. Proportions (*y*-axis) of cell subsets (color legend) across high and low S100 score groups. (E) The cell proportions comparison in each tumor sample between high and low S100 score groups.

A detailed characterization of the immune microenvironment was visualized using a tSNE plot (Fig. 5A). To illustrate the distribution of different cell clusters between high and low S100 score groups, we generated bar plots showcasing the distribution proportions of cell clusters from high S100 score samples to low S100 score samples (Fig. 5B–D). Utilizing the Wilcoxon test, we compared immune cell infiltration differences between high and low S100 score groups, highlighting the statistical significance in malignant cells, CD4^+^ T cells, CD8^+^ T cells, and NK cells.

Notably, we observed differences in CD8^+^ T cells, CD4^+^ T cells, and NK cells between high and low S100 score groups. However, the signaling pathway network and ligand-receptor pairs between malignant cells and immune cells remained unknown, prompting our exploration of these network interactions.

**Figure 6:**
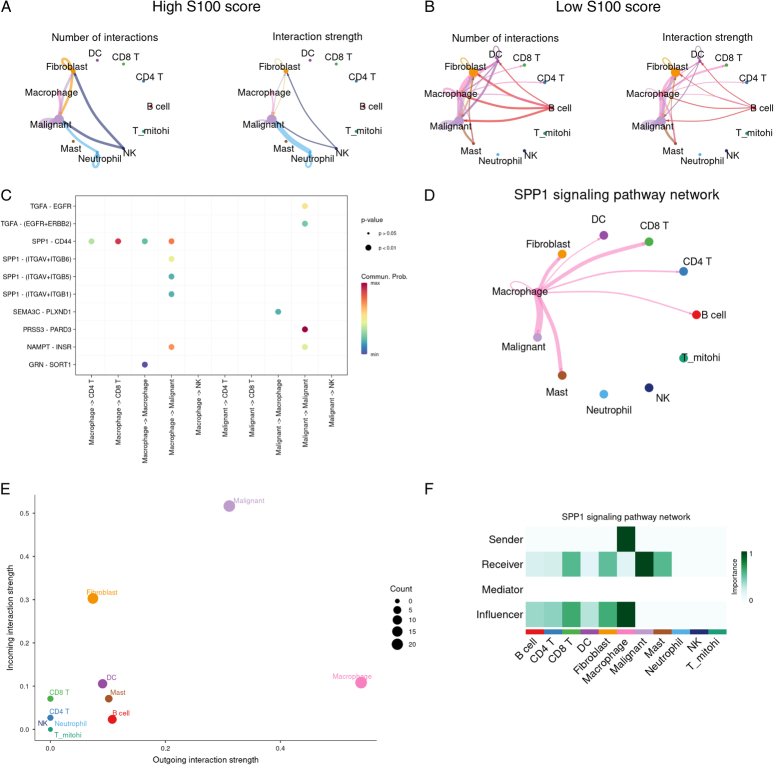
CellChat analysis between malignant cells and T cells. (A) Number and interaction strength of significant ligand-receptor pairs between any pair of two cell populations in the high S100 score group. (B) Number and interaction strength of significant ligand-receptor pairs between any pair of two cell populations in the low S100 score group. (C) Comparison of the significant ligand-receptor pairs between T cells and other cells in the low S100 score group. Dot color reflects communication probabilities and dot size represents computed *P*-values. (D) SPP1 signaling pathway network between any pair of two cell populations in the low S100 score group. Circle sizes are proportional to the number of cells in each cell group and edge width represents the communication probability. (E) The dot plot showing the comparison of outgoing and incoming signaling patterns of all cell types in low S100 score group. The dot size is proportional to the contribution score computed from pattern recognition analysis. (F) The interaction role of all cell types in the SPP1 signaling pathway network.

Employing the R package CellChat, we explored the signaling pathway network and ligand-receptor pairs between two cell populations. We displayed the number and interaction strength of significant ligand-receptor pairs between any pair of two cell populations in both high and low S100 score groups (Fig. 6A–B). Our focus was directed towards the interaction signaling pathway network between CD8^+^ T cells, CD4^+^ T cells, NK cells, and other cells. Our findings identified SEMA signaling as key pathway with the receptor PLXND1 between malignant cells and macrophage cells. In the low S100 score group, macrophage cells were found to modulate CD8^+^ and CD4^+^ T cells indirectly through the SPP1 pathway signaling, utilizing the receptor CD44 (Fig. 6C). We further presented the SPP1 signaling pathway network between macrophage cells and other cells, including CD8^+^ and CD4^+^ T cells, in low S100 score group (Fig. 6D). Additionally, we revealed outgoing and incoming interaction strengths and the interaction roles of all cell types in the SPP1 signaling pathway network (Fig. 6E–F).

While our results provided accurate insights into malignant progression, changes in immune cell infiltration, and tumor microenvironment interactions networks at the single-cell level driven by S100 genes, we acknowledge the absence of consideration for therapy-associated clinical features. Consequently, our future exploration will focus on identifying the core S100 gene as a biomarker or neoadjuvant sensitivity.

### Change of S100 score-associated neoadjuvant sensitivity and exploration of core S100 gene drug resistance with experimental verification

**Figure 7:**
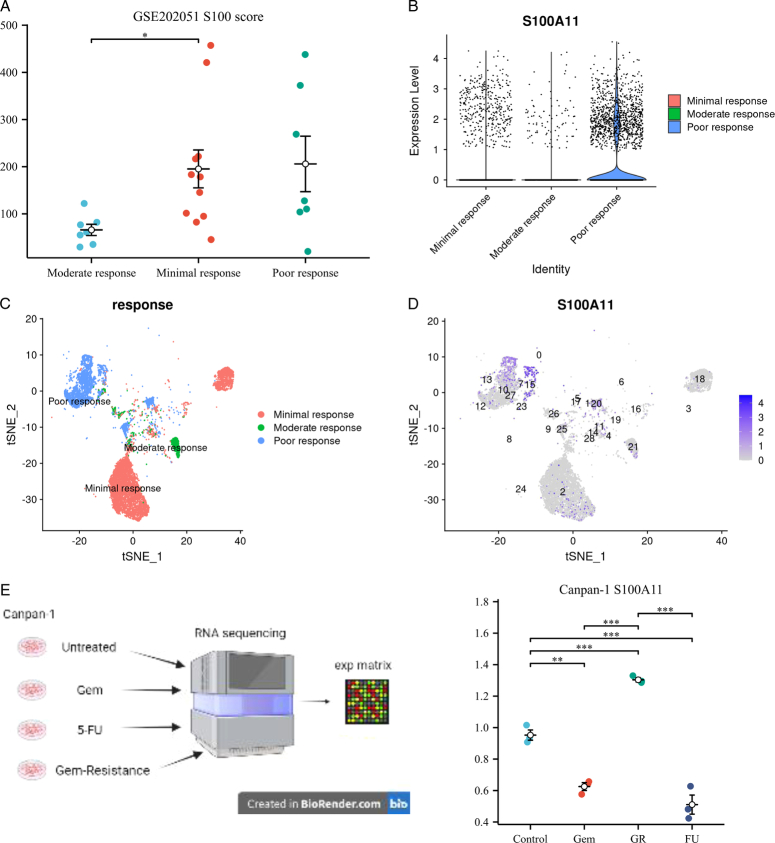
The S100 family members as a biomarker of neoadjuvant chemotherapy response. The comparison of S100 score between different treatment responses. (B–D) The expression level of S100A11 in different treatment responses. (E) The comparison of S100A11 between control, Gem,5-FU, and Gem-Resistance of Canpan-1 cell lines.

We conducted a Wilcoxon test to compare S100 scores among groups with varying responses to neoadjuvant chemotherapy, specifically moderate response, minimal response, and poor response. The results, illustrated in Figure 7A, clearly demonstrated an increase in S100 scores associated with poorer treatment response. Further exploration led us to identify S100A11 as the core gene underlying sensitivity changes. By analyzing the expression levels of S100 genes at the single-cell level across different treatment response groups, we found that in poor responders to gemcitabine, only the expression level of S100A11 was significantly higher (Figure S5, Supplemental Digital Content 10, http://links.lww.com/JS9/C181) (Fig. 7B–D). We identified the treatment concentration of gemcitabine and 5-FU of pancreatic cancer cell lines with IC50 and also provided the IC50 of gemcitabine-resistance cell lines (Figure S6, Supplemental Digital Content 11, http://links.lww.com/JS9/C182). To validate the role of S100A11 as a core gene in drug resistance, we cultured pancreatic cells under various conditions, including normal media, gemcitabine-treated, 5-FU-treated, and gemcitabine drug-resistant. The samples obtained from these cultures were subjected to RNA sequencing. Employing the Wilcoxon test, we compared S100A11 expression levels across different treatment groups, providing further evidence of the significance of S100A11 as the core gene associated with drug resistance (Fig. 7E).

## Discussion

The role of S100 family members in various components of the tumor microenvironment, including tumor cells, cancer-associated fibroblasts (CAFs), immune cells, and immune checkpoint molecules, has opened new avenues for the development of novel therapeutic strategies. Padilla and colleagues have demonstrated the significant involvement of extracellular S100A7 protein in tumor cell migration and invasion, as well as in the recruitment and differentiation of immune cells. The release of S100A7 into the bloodstream, contributed to the formation of a metastatic niche, supporting tumor metastasis. Their findings suggest that S100A7 protein could serve as a potential therapeutic target, for cancer, with monoclonal antibodies extracellular S100A7 showing promise^38^. Additionally, Gao *et al*.^39^ explored the antiglioma properties of Dul, a S100B inhibitor, by shifting tumor-associated macrophages (TAMs) into proinflammatory subtypes, indicating the potential for enhanced antitumor efficacy when combined with immunotherapy. Wagner *et al*.^40^ reported that the tumor microenvironment-derived protein S100A8/A9 served as a predicative biomarker for melanoma patients undergoing immunotherapy with anti-PD-1 antibodies, underscoring the importance of S100 family members as suitable biomarkers. However, the existing literature on S100 genes prognosis and mechanisms is characterized by disorderly and unsystematic research, resulting in a lack of distinct and uniform interpretations of the prognostic significance of S100 genes.

There are standardized application values and innovative points in our research. The study selected GSE71729 cohorts as training cohorts because the cohort contain 125 primary pancreatic adenocarcinoma samples with clinical follow-up information besides 16 pancreatic cancer cell lines, 46 normal pancreatic tissue sample and 12 types of metastasis samples. The cohort was convenient to provide phenotypes prediction analysis for exploring cell experiments and metastasis mechanism. Any prediction model makes a good model to be characterized the two related properties of discrimination and calibration^41^. Researchers usually use the receiver operating characteristic (ROC) curve or C statistic to characterize discrimination of survival outcomes^42^. The calibration curve can provide two indexes to characterize the calibration of model^43^.

Therefore, we provided ROC curves and calibration to identify the efficacy of prediction model. In general, 1-year prediction AUC of AJCC TNM was lower than 0.6^44,45^. One year prediction AUC of our model was 0.654 and 2-year and 4-year prediction AUCs presented high value (0.779 and 0.830). Other RNA sequence cohorts contained expression matrices and clinical follow-up information and were applied for external validation. TCGA and GSE79668 cohorts contained TNM stage information, age, and sex besides clinical follow-up information. After external validation, our study selected the two cohorts to analyze univariate and multivariate cox regression results of S100 subgroup and display subgroup as independent prognostic factor.

In consideration of pancreatic specific anatomical site, the difficulty and low specificity of tumor tissue acquisition leaded to a variety of limitations^11^. The conventional serum biomarkers CEA and CA 19-9 provided the single diagnosis efficacy for the degree of malignancy^12–14^. There were lack of effective methods of the identification of high risk group with poor response for therapy. The S100 gene family members were reported by many study teams associated with pancreatic adenocarcinoma malignant progression. Taken into consideration this practical application, we provided the nomogram RiskScore model to optimize the S100 score prediction system and the RiskScore model integrated age and N stage to be possible to provide better prediction efficacy for high risk group in TCGA cohort. Because there was the lack of external validation in integrated RiskScore model, practical clinical application was worthy of discussion.

Intrinsically, there is a complex interactive clinical and molecular hierarchy of different layers within one tumor sample^46^. Tang *et al*.^47^ analyzed the comprehension matrix of single-cell and spatial transcriptomes, proteomes, bulk transcriptomes, metabolomes, and metabolic flux and discovered neoadjuvant chemotherapy downregulated glycolysis and upregulated CD36 expression. After construction of S100 prognosis model, we integrated and analyzed bulk transcriptomes and single-cell transcriptomes with multiple R packages to reveal the metabolism pathway of malignant progression and immune microenvironment change. Our study results drove clinical and basic translational research of pancreatic cancer surgery.

Over the years, metabolic reprogramming has emerged as a crucial node in cancer hallmarks, influencing cancer cell proliferation, metastasis, and resistance to therapies^48^. Purines and pyrimidines are salvaged deoxyribonucleotides synthesis,with these serving as raw materials for DNA synthesis^49^. Pyrimidine antimetabolites such as gemcitabine and 5-FU are nowadays extensively used in pancreatic adenocarcinoma chemotherapy, modulated by enzymes in the pyrimidine metabolic pathway to transform into analogs of cellular nucleotides. This biological process inhibits DNA synthesis, inducing DNA damage and apoptosis in cancer cells^50^. The elevated expression levels of S100 genes in pancreatic adenocarcinoma enhanced pyrimidine metabolism, necessitating the labeling of U^13^-C glucose for exploring the target enzymes modulated by S100 genes in de novo synthesis. Utilizing metabolomics technology, we sought to illustrate changes in car-aspartate and dihydroorotate content in the pathway. Furthermore, we propose culturing patient-derived xenografts, categorizing samples based on the S100 score model, and comparing tumor growth and pyrimidine metabolism levels between groups.

It has been established that tumor microenvironment comprised of malignant cells, fibroblast cells, immune cells, and their secreted molecules, play pivotal roles in cancer progression^51^. Our research identified pyrimidine metabolism as a key pathway in S100-driven malignant potential. To further understand this, we compared the proportion of immune cell infiltration between high and low S100 score. Our results revealed significant changes in CD8^+^ T cells, CD4^+^ T cells, and NK cells. Among these, CD8^+^ T cells emerged as particularly potent immune effector cells, utilizing T cell receptors recognized by cancer cells and forming complexes with MHC-peptide complexes from cancer cells^52^. Subsequently, CD8^+^ T cells induced apoptosis through perforin and granzyme, displaying antitumor cytotoxicity^53^. The Th1 subtype of CD4^+^ T cells played a supportive role by producing IFNγand TNF-α, aiding the cytotoxic activity of CD8^+^ T cells^54^. The synchronous decrease of CD4^+^ T cells, driven by S100 genes, suggests that the Th1 subtype may play a pivotal role in the immune microenvironment of pancreatic adenocarcinoma. Notably, NK cells, known for their anticancer potential, are gaining attention for cellular therapies^55^. In our study, we observed a reduction in three types of immune cells in the low S100 score group, but ligand-receptor interactions between malignant cells and immune cells were not explored.

To comprehensively analyze intercellular communications, we employed CellChat, a tool that integrates single-cell RNA sequencing data with the CellChatDB signaling molecule interaction database^31^. Our study revealed that macrophages serve as intermediate cells, supplying functional molecules to both malignant cells and T cells. A previous investigation demonstrated a positive correlation between SPP1^+^ tumor-associated macrophages and colorectal cancer progression^56^. However, our findings indicated higher levels of SPP1 expression in the macrophage group within the low S100 score category, associated with a better prognosis. Macrophage cells, equipped with the SPP1 ligand, activated malignant cell via the CD44 receptor, subsequently inducing CD8^+^ and CD4^+^ T cells to transform into effector T cells. We hypothesized that malignant cells with lower potential malignancy potential might exhibit sensitivity to the SPP1-CD44 pathway, thereby influencing the T cell microenvironment. Consequently, a high infiltration of T cells in the low S100 score group disrupted the immune suppressive state in pancreatic adenocarcinoma. To validate the significance of this pathway, experimental research, particularly utilizing flow cytometry technology, is planned to isolate SPP1+ macrophages in low S100 score samples and offer supplementary verification for CD44 receptor expression in CD8^+^ and CD4^+^ T cells.

Pancreatic adenocarcinoma can be categorized into resectable, borderline resectable, locally advanced, and metastatic types^57^. For resectable cases, surgery followed by adjuvant chemotherapy is the primary treatment choice^58^. Adjuvant combination therapies involving albumin-bound paclitaxel with gemcitabine or FOLFIRINOX have shown improved improve overall survival compared to surgery alone^59–61^. The chemotherapy, with or without targeted therapy, is the initial approach for unresectable pancreatic adenocarcinoma^62^. However, chemotherapy resistance is a prevalent issue, garnering significant attention from researchers^63^. The chemotherapy agents mentioned above are pyrimidine antimetabolites that function intracellularly by traversing the plasma membrane. It is now understood that the cellular uptake of these molecules is mediated by various nucleoside transporters^64^. Recent studies have focused on the modulation of nucleoside transporters in pancreatic adenocarcinoma, with emphasis on the potential involvement of S100A11^65^. It is highly conceivable that S100A11 participates in this process. To substantiate this hypothesis, our research plan involves conducting experiments utilizing immunoblots, reverse transcription polymerase chain reaction (RT-PCR), and liquid chromatography tandem mass spectrometry. These experiments aim to validate and establish the correlation between S100A11 and the genes associated with nucleotide transporters.

However, there are many limitations to our research. The training cohort GSE71729 do not contain sex, age, and TNM staging. We cannot provide Cox regression results of S100 subgroup in training cohort. As for the prediction of neoadjuvant therapy response, only GSE202051 cohort identified the poor response prediction efficacy. There was the lack of in-house pancreatic adenocarcinoma samples cohort construction including RNA sequence matrix, clinical follow-up information, other basic information including sex, age, and TNM staging, and chemotherapy response data and we should use the cohort to validate our prediction model. The progression of tumor activated metabolic pathways of cancer cells, contributed to a nutrient-depleted and hypoxic microenvironment and remodeled vasculature within the TME and established metabolic competition between cancer cells and infiltrating immune cells^66^. The S100 family genes drove tumor progression to activate pyrimidine metabolism pathway and change immune cells infiltration proportion in microenvironment. There were the lack of metabolomics sequencing and flow cytometry detection to identify metabolites and immune microenvironment change.

## Conclusion

The study provided six S100 genes from family members to construct prognosis prediction model with better discrimination and calibration, explained pyrimidine metabolites change as malignant progression mechanism of model genes and malignant progression driving macrophage cells as intermediate to change effective immune cells proportion including CD8^+^T cells and CD4^+^T cells in single-cell levels. S100A11 was screened as probable biomarker of neoadjuvant therapy and worthy to be studied for experimental research.

## Ethical approval

Not involving with patients.

## Consent

Not involving with patients.

## Sources of funding

This work was supported by grants from the Municipal Health Commission (201940019), National Natural Science Foundation of China (82273382, 81827807、81972218、81972257、82103409、82272929、82173116), Program of Shanghai Academic/Technology Research Leader (23XD1400600), China Postdoctoral Science Foundation (2021M690037), Shanghai Sailing Program (21YF1407100), Science and Technology Planning Project of Yunnan Province (202305AF150148), and Shanghai Municipal Health Commission (20224Y0307). The funding agencies had no role in the study design, data collection and analyses, decision to publish, or preparation of the manuscript.

## Author contribution

Z.-J.X., J.-A.L., and Z.-Y.C.: collected the data acquisition, provided technical support, and drafted the manuscript drafting; Z.-J.X., Y.Y., Z.-H.X., C.S., R.-J.L., and Y.G.: designed the experimental protocol and conduct the experiments; H.-X.X., W.-Q.W., and L.L.: sought funding for the research, developed the study design, and supervised the study. All authors read and approved the final manuscript.

## Conflicts of interest disclosure

We have no conflicts of interest to declare.

## Research registration unique identifying number (UIN)

No. Our research was not clinical trials.

## Guarantor

Zi-Jin Xu, Department of Pancreatic Surgery, Zhongshan Hospital, Fudan University, Shanghai, China. Liang Liu, Department of Pancreatic Surgery, Zhongshan Hospital, Fudan University, Shanghai, China.

## Data availability statement

Yes.

## Provenance and peer review

Not commissioned, externally peer-reviewed.

## Supplementary Material

**Figure:**
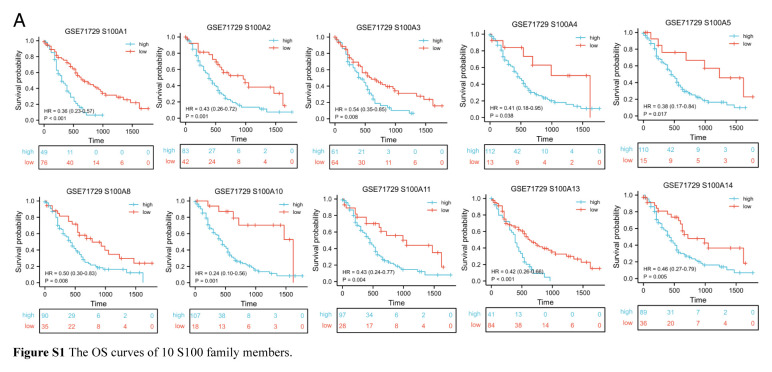


**Figure:**
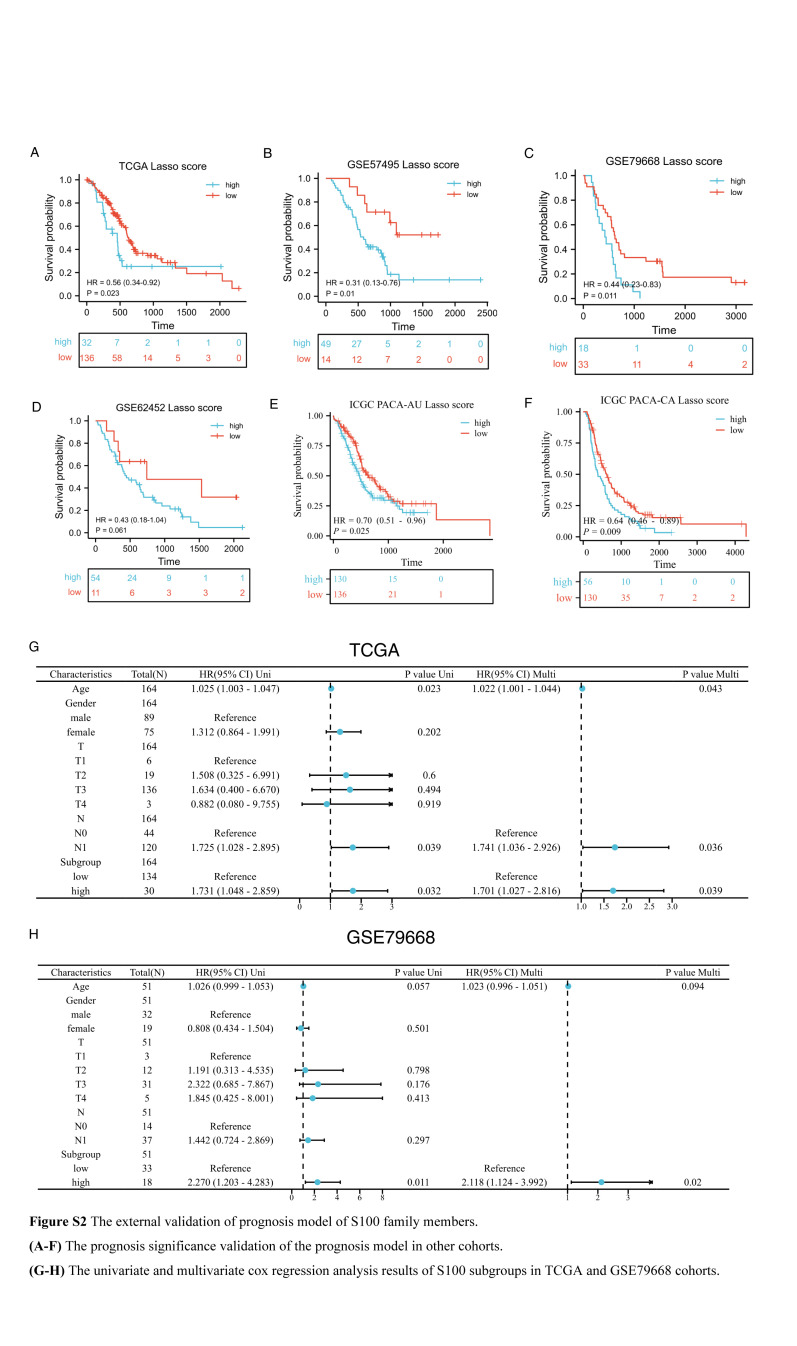


**Figure:**
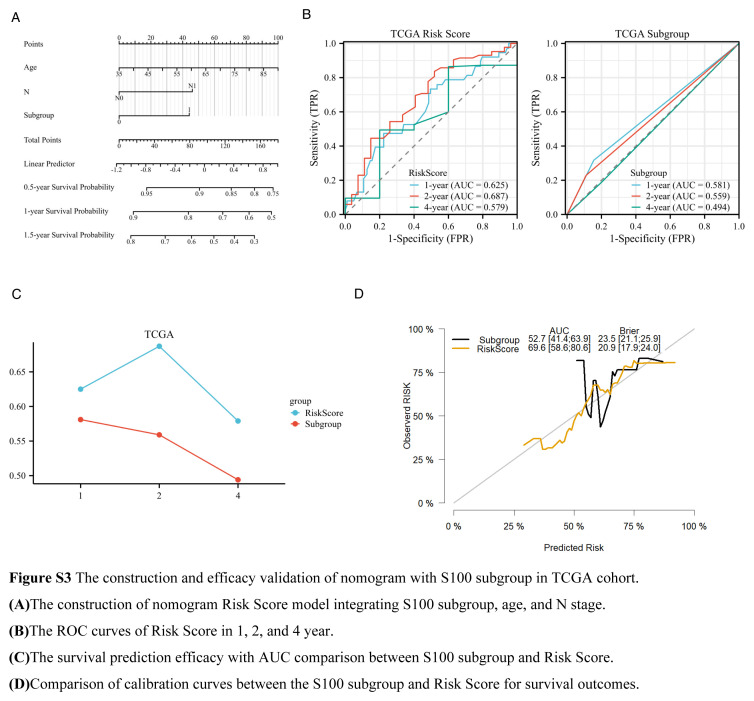


**Figure:**
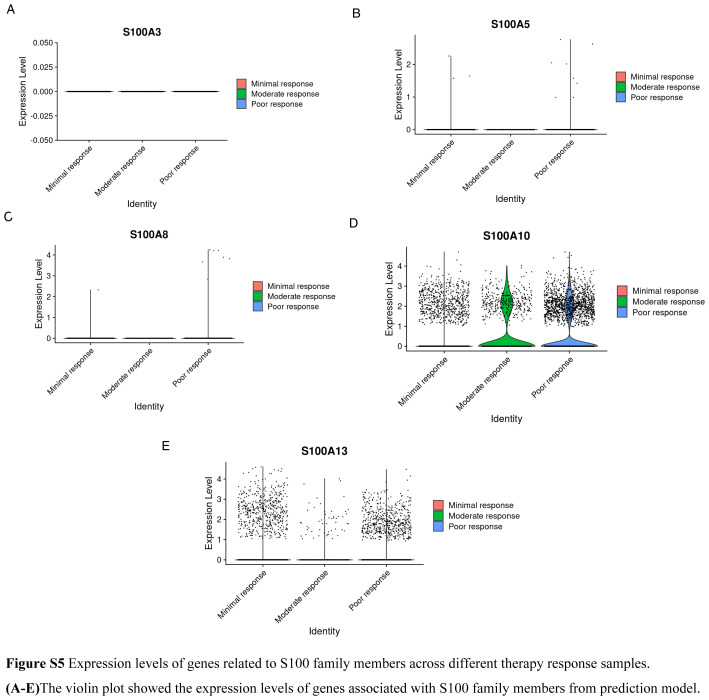


**Figure:**
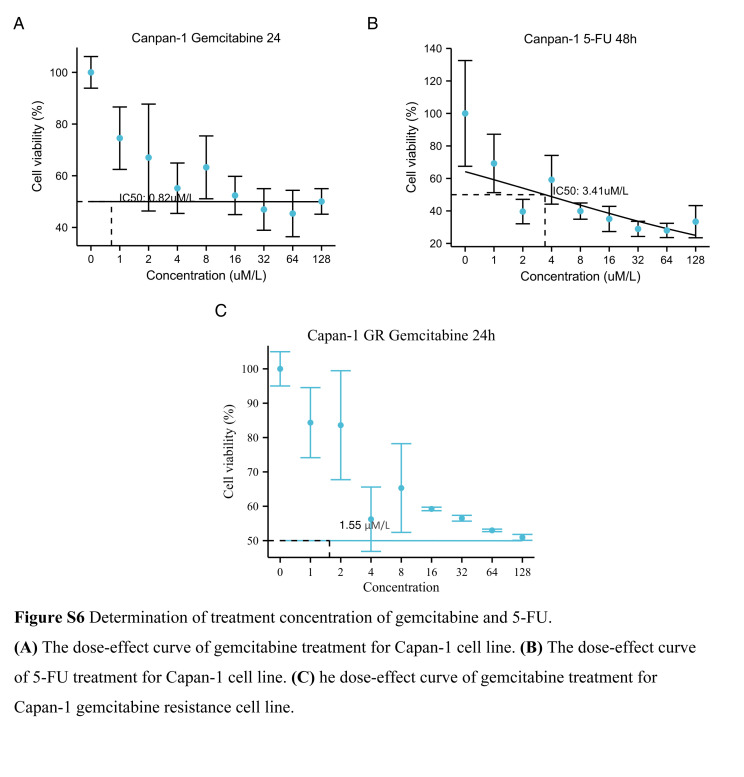


## References

[1] Gaddam S Abboud Y Oh J. Incidence of pancreatic cancer by age and sex in the US, 2000-2018. Jama 2021;326:2075–2077. 34689206 10.1001/jama.2021.18859 PMC8543346

[2] Siegel RL Miller KD Fuchs HE. Cancer statistics, 2022. CA Cancer J Clin 2022;72:7–33. 35020204 10.3322/caac.21708

[3] Millikan KW Deziel DJ Silverstein JC. Prognostic factors associated with resectable adenocarcinoma of the head of the pancreas. Am Surg 1999;65:618–623. 10399969

[4] Oettle H Neuhaus P Hochhaus A. Adjuvant chemotherapy with gemcitabine and long-term outcomes among patients with resected pancreatic cancer: the CONKO-001 randomized trial. Jama 2013;310:1473–1481. 24104372 10.1001/jama.2013.279201

[5] Versteijne E Vogel JA Besselink MG. Meta-analysis comparing upfront surgery with neoadjuvant treatment in patients with resectable or borderline resectable pancreatic cancer. Br J Surg 2018;105:946–958. 29708592 10.1002/bjs.10870 PMC6033157

[6] Bruni D Angell HK Galon J. The immune contexture and Immunoscore in cancer prognosis and therapeutic efficacy. Nat Rev Cancer 2020;20:662–680. 32753728 10.1038/s41568-020-0285-7

[7] Ho WJ Jaffee EM Zheng L. The tumour microenvironment in pancreatic cancer - clinical challenges and opportunities. Nat Rev Clin Oncol 2020;17:527–540. 32398706 10.1038/s41571-020-0363-5 PMC7442729

[8] Danilova L Ho WJ Zhu Q. Programmed Cell Death Ligand-1 (PD-L1) and CD8 expression profiling identify an immunologic subtype of pancreatic ductal adenocarcinomas with favorable survival. Cancer Immunol Res 2019;7:886–895. 31043417 10.1158/2326-6066.CIR-18-0822 PMC6548624

[9] Collisson EA Bailey P Chang DK. Molecular subtypes of pancreatic cancer. Nat Rev Gastroenterol Hepatol 2019;16:207–220. 30718832 10.1038/s41575-019-0109-y

[10] Socinski MA Jotte RM Cappuzzo F. Atezolizumab for first-line treatment of metastatic nonsquamous NSCLC. N Engl J Med 2018;378:2288–2301. 29863955 10.1056/NEJMoa1716948

[11] Qi ZH Xu HX Zhang SR. The significance of liquid biopsy in pancreatic cancer. J Cancer 2018;9:3417–3426. 30271504 10.7150/jca.24591 PMC6160675

[12] Goonetilleke KS Siriwardena AK. Systematic review of carbohydrate antigen (CA 19-9) as a biochemical marker in the diagnosis of pancreatic cancer. Eur J Surg Oncol 2007;33:266–270. 17097848 10.1016/j.ejso.2006.10.004

[13] Gattani AM Mandeli J Bruckner HW. Tumor markers in patients with pancreatic carcinoma. Cancer 1996;78:57–62. 8646727 10.1002/(SICI)1097-0142(19960701)78:1<57::AID-CNCR10>3.0.CO;2-6

[14] Ni XG Bai XF Mao YL. The clinical value of serum CEA, CA19-9, and CA242 in the diagnosis and prognosis of pancreatic cancer. Eur J Surg Oncol 2005;31:164–169. 15698733 10.1016/j.ejso.2004.09.007

[15] Bresnick AR Weber DJ Zimmer DB. S100 proteins in cancer. Nat Rev Cancer 2015;15:96–109. 25614008 10.1038/nrc3893 PMC4369764

[16] Allgöwer C Kretz AL von Karstedt S. Friend or Foe: S100 proteins in cancer. Cancers (Basel) 2020;12:2037. 32722137 10.3390/cancers12082037 PMC7465620

[17] Xia C Braunstein Z Toomey AC. S100 proteins as an important regulator of macrophage inflammation. Front Immunol 2017;8:1908. 29379499 10.3389/fimmu.2017.01908 PMC5770888

[18] Ji YF HUANG H JIANG F. S100 family signaling network and related proteins in pancreatic cancer (Review). Int J Mol Med 2014;33:769–776. 24481067 10.3892/ijmm.2014.1633

[19] Moffitt RA Marayati R Flate EL. Virtual microdissection identifies distinct tumor- and stroma-specific subtypes of pancreatic ductal adenocarcinoma. Nat Genet 2015;47:1168–1178. 26343385 10.1038/ng.3398 PMC4912058

[20] Chen DT Davis-Yadley AH Huang PY. Prognostic fifteen-gene signature for early stage pancreatic ductal adenocarcinoma. PLoS One 2015;10:e0133562. 26247463 10.1371/journal.pone.0133562 PMC4527782

[21] Kirby MK Ramaker RC Gertz J. RNA sequencing of pancreatic adenocarcinoma tumors yields novel expression patterns associated with long-term survival and reveals a role for ANGPTL4. Mol Oncol 2016;10:1169–1182. 27282075 10.1016/j.molonc.2016.05.004 PMC5423196

[22] Yang S He P Wang J. A novel MIF signaling pathway drives the malignant character of pancreatic cancer by targeting NR3C2. Cancer Res 2016;76:3838–3850. 27197190 10.1158/0008-5472.CAN-15-2841 PMC4930741

[23] Hwang WL Jagadeesh KA Guo JA. Single-nucleus and spatial transcriptome profiling of pancreatic cancer identifies multicellular dynamics associated with neoadjuvant treatment. Nat Genet 2022;54:1178–1191. 35902743 10.1038/s41588-022-01134-8 PMC10290535

[24] Tibshirani R. Regression shrinkage and selection via the Lasso. J Royal Statist Society: Series B (Methodological) 1996;58:267–288.

[25] Fawcett T. An introduction to ROC analysis. Pattern Recognition Letters 2006;27:861–874.

[26] Hosmer DW HOSMER T LE CESSIE S. A comparison of goodness-of-fit tests for the logistic regression model. Stat Med 1997;16:965–980. 9160492 10.1002/(sici)1097-0258(19970515)16:9<965::aid-sim509>3.0.co;2-o

[27] Satija R Farrell JA Gennert D. Spatial reconstruction of single-cell gene expression data. Nat Biotechnol 2015;33:495–502. 25867923 10.1038/nbt.3192 PMC4430369

[28] Ritchie ME Phipson B Wu D. limma powers differential expression analyses for RNA-sequencing and microarray studies. Nucleic Acids Res 2015;43:e47. 25605792 10.1093/nar/gkv007 PMC4402510

[29] Subramanian A Tamayo P Mootha VK. Gene set enrichment analysis: a knowledge-based approach for interpreting genome-wide expression profiles. Proc Natl Acad Sci U S A 2005;102:15545–15550. 16199517 10.1073/pnas.0506580102 PMC1239896

[30] Efremova M Vento-Tormo M Teichmann SA. CellPhoneDB: inferring cell-cell communication from combined expression of multi-subunit ligand-receptor complexes. Nat Protoc 2020;15:1484–1506. 32103204 10.1038/s41596-020-0292-x

[31] Jin S Guerrero-Juarez CF Zhang L. Inference and analysis of cell-cell communication using CellChat. Nat Commun 2021;12:1088. 33597522 10.1038/s41467-021-21246-9 PMC7889871

[32] Altman DG McShane LM Sauerbrei W. Reporting recommendations for tumor marker prognostic studies (REMARK): explanation and elaboration. BMC Med 2012;10:51. 22642691 10.1186/1741-7015-10-51 PMC3362748

[33] Wu Y Zhou Q Guo F. S100 proteins in pancreatic cancer: current knowledge and future perspectives. Front Oncol 2021;11:711180. 34527585 10.3389/fonc.2021.711180 PMC8435722

[34] Loffler M Fairbanks L Zameitat E. Pyrimidine pathways in health and disease. Trends Mol Med 2005;11:430–437. 16098809 10.1016/j.molmed.2005.07.003

[35] Neoptolemos JP Palmer DH Ghaneh P. Comparison of adjuvant gemcitabine and capecitabine with gemcitabine monotherapy in patients with resected pancreatic cancer (ESPAC-4): a multicentre, open-label, randomised, phase 3 trial. Lancet 2017;389:1011–1024. 28129987 10.1016/S0140-6736(16)32409-6

[36] Neoptolemos JP Moore MJ Cox TF. Effect of adjuvant chemotherapy with fluorouracil plus folinic acid or gemcitabine vs observation on survival in patients with resected periampullary adenocarcinoma: the ESPAC-3 periampullary cancer randomized trial. Jama 2012;308:147–156. 22782416 10.1001/jama.2012.7352

[37] Singh P Ali SA. Multifunctional role of S100 protein family in the immune system: an update. Cells 2022;11:2274. 35892571 10.3390/cells11152274 PMC9332480

[38] Padilla L Dakhel S Adan J. S100A7: from mechanism to cancer therapy. Oncogene 2017;36:6749–6761. 28825725 10.1038/onc.2017.283

[39] Gao H Zhang IY Zhang L. S100B suppression alters polarization of infiltrating myeloid-derived cells in gliomas and inhibits tumor growth. Cancer Lett 2018;439:91–100. 30076898 10.1016/j.canlet.2018.07.034 PMC7048242

[40] Wagner NB Weide B Gries M. Tumor microenvironment-derived S100A8/A9 is a novel prognostic biomarker for advanced melanoma patients and during immunotherapy with anti-PD-1 antibodies. J Immunother Cancer 2019;7:343. 31806053 10.1186/s40425-019-0828-1 PMC6896585

[41] Alba AC Agoritsas T Walsh M. Discrimination and calibration of clinical prediction models: users’ guides to the medical literature. Jama 2017;318:1377–1384. 29049590 10.1001/jama.2017.12126

[42] Pencina MJ D’Agostino RB Sr. Evaluating discrimination of risk prediction models: the C statistic. Jama 2015;314:1063–1064. 26348755 10.1001/jama.2015.11082

[43] Van Calster B Nieboer D Vergouwe Y. A calibration hierarchy for risk models was defined: from utopia to empirical data. J Clin Epidemiol 2016;74:167–176. 26772608 10.1016/j.jclinepi.2015.12.005

[44] Zou W Wang Z Wang F. A nomogram predicting overall survival in patients with non-metastatic pancreatic head adenocarcinoma after surgery: a population-based study. BMC Cancer 2021;21:524. 33964898 10.1186/s12885-021-08250-4 PMC8106852

[45] Wu M Li X Zhang T. Identification of a nine-gene signature and establishment of a prognostic nomogram predicting overall survival of pancreatic cancer. Front Oncol 2019;9:996. 31612115 10.3389/fonc.2019.00996 PMC6776930

[46] Regev A Teichmann SA Lander ES. The human cell atlas. Elife 2017;6:e27041. 29206104 10.7554/eLife.27041 PMC5762154

[47] Tang R Xu J Wang W. Targeting neoadjuvant chemotherapy-induced metabolic reprogramming in pancreatic cancer promotes anti-tumor immunity and chemo-response. Cell Rep Med 2023;4:101234. 37852179 10.1016/j.xcrm.2023.101234 PMC10591062

[48] Zou S Qin B Yang Z. CSN6 mediates nucleotide metabolism to promote tumor development and chemoresistance in colorectal cancer. Cancer Res 2023;83:414–427. 36512632 10.1158/0008-5472.CAN-22-2145

[49] Parker WB. Enzymology of purine and pyrimidine antimetabolites used in the treatment of cancer. Chem Rev 2009;109:2880–2893. 19476376 10.1021/cr900028p PMC2827868

[50] Sampath D Rao VA Plunkett W. Mechanisms of apoptosis induction by nucleoside analogs. Oncogene 2003;22:9063–9074. 14663485 10.1038/sj.onc.1207229

[51] Bejarano L Jordāo MJC Joyce JA. Therapeutic targeting of the tumor microenvironment. Cancer Discov 2021;11:933–959. 33811125 10.1158/2159-8290.CD-20-1808

[52] Philip M Schietinger A. CD8 (+) T cell differentiation and dysfunction in cancer. Nat Rev Immunol 2022;22:209–223. 34253904 10.1038/s41577-021-00574-3 PMC9792152

[53] Zheng L Qin S Si W. Pan-cancer single-cell landscape of tumor-infiltrating T cells. Science 2021;374:abe6474. 34914499 10.1126/science.abe6474

[54] DeNardo DG Barreto JB Andreu P. CD4 (+) T cells regulate pulmonary metastasis of mammary carcinomas by enhancing protumor properties of macrophages. Cancer Cell 2009;16:91–102. 19647220 10.1016/j.ccr.2009.06.018 PMC2778576

[55] Chan IS Ewald AJ. The changing role of natural killer cells in cancer metastasis. J Clin Invest 2022;132:e143762. 35289318 10.1172/JCI143762 PMC8920322

[56] Qi J Sun H Zhang Y. Single-cell and spatial analysis reveal interaction of FAP(+) fibroblasts and SPP1 (+) macrophages in colorectal cancer. Nat Commun 2022;13:1742. 35365629 10.1038/s41467-022-29366-6 PMC8976074

[57] Yu S Zhang C Xie KP. Therapeutic resistance of pancreatic cancer: Roadmap to its reversal. Biochim Biophys Acta Rev Cancer 2021;1875:188461. 33157162 10.1016/j.bbcan.2020.188461

[58] Dumont R Puleo F Collignon J. A single center experience in resectable pancreatic ductal adenocarcinoma: the limitations of the surgery-first approach. Critical review of the literature and proposals for practice update. Acta Gastroenterol Belg 2017;80:451–461. 29560639

[59] Oettle H Post S Neuhaus P. Adjuvant chemotherapy with gemcitabine vs observation in patients undergoing curative-intent resection of pancreatic cancer: a randomized controlled trial. Jama 2007;297:267–277. 17227978 10.1001/jama.297.3.267

[60] Neoptolemos JP Stocken DD Bassi C. Adjuvant chemotherapy with fluorouracil plus folinic acid vs gemcitabine following pancreatic cancer resection: a randomized controlled trial. Jama 2010;304:1073–1081. 20823433 10.1001/jama.2010.1275

[61] Conroy T Hammel P Hebbar M. FOLFIRINOX or gemcitabine as adjuvant therapy for pancreatic cancer. N Engl J Med 2018;379:2395–2406. 30575490 10.1056/NEJMoa1809775

[62] Conroy T Desseigne F Ychou M. FOLFIRINOX versus gemcitabine for metastatic pancreatic cancer. N Engl J Med 2011;364:1817–1825. 21561347 10.1056/NEJMoa1011923

[63] Zeng S Pöttler Lan. Chemoresistance in pancreatic cancer. Int J Mol Sci 2019;20:4504. 31514451 10.3390/ijms20184504 PMC6770382

[64] Rauchwerger DR Firby PS Hedley DW. Equilibrative-sensitive nucleoside transporter and its role in gemcitabine sensitivity. Cancer Res 2000;60:6075–6079. 11085530

[65] Mackey JR Mani RS Selner M. Functional nucleoside transporters are required for gemcitabine influx and manifestation of toxicity in cancer cell lines. Cancer Res 1998;58:4349–4357. 9766663

[66] Leone RD Powell JD. Metabolism of immune cells in cancer. Nat Rev Cancer 2020;20:516–531. 32632251 10.1038/s41568-020-0273-y PMC8041116

