# Supplementary material for: Construction of S100 family members prognosis prediction model and analysis of immune microenvironment landscape at single-cell level in pancreatic adenocarcinoma: a tumor marker prognostic study
Source: Int J Surg. 2024 Mar 18;110(6):3591–605. doi: 10.1097/JS9.0000000000001293 (PMC11175822; doi:10.1097/JS9.0000000000001293)
Supplement: Supplementary file 5 [file js9-110-3591-s008.docx]

**Table S4** The index of nomogram prediction model accuracy degree in TCGA cohort.

| 0.5-year survival prediction |  | True | |
| --- | --- | --- | --- |
|  |  | Positive | Negative |
| Prediction | Positive | 62 | 6 |
|  | Negative | 77 | 19 |
| 1-year survival prediction | True | | |
|  |  | Positive | Negative |
| Prediction | Positive | 50 | 18 |
|  | Negative | 54 | 42 |

0.5-year survival prediction

ACC=0.494

PPV=0.912

Sensitivity=0.446

Specificity=0.76

NPV=0.198

1-year survival prediction

ACC=0.561

PPV=0.735

Sensitivity=0.481

Specificity=0.7

NPV=0.438
