# Supplementary figures and images for: Construction of S100 family members prognosis prediction model and analysis of immune microenvironment landscape at single-cell level in pancreatic adenocarcinoma: a tumor marker prognostic study
Source: Int J Surg. 2024 Mar 18;110(6):3591–605. doi: 10.1097/JS9.0000000000001293 (PMC11175822; doi:10.1097/JS9.0000000000001293)

A

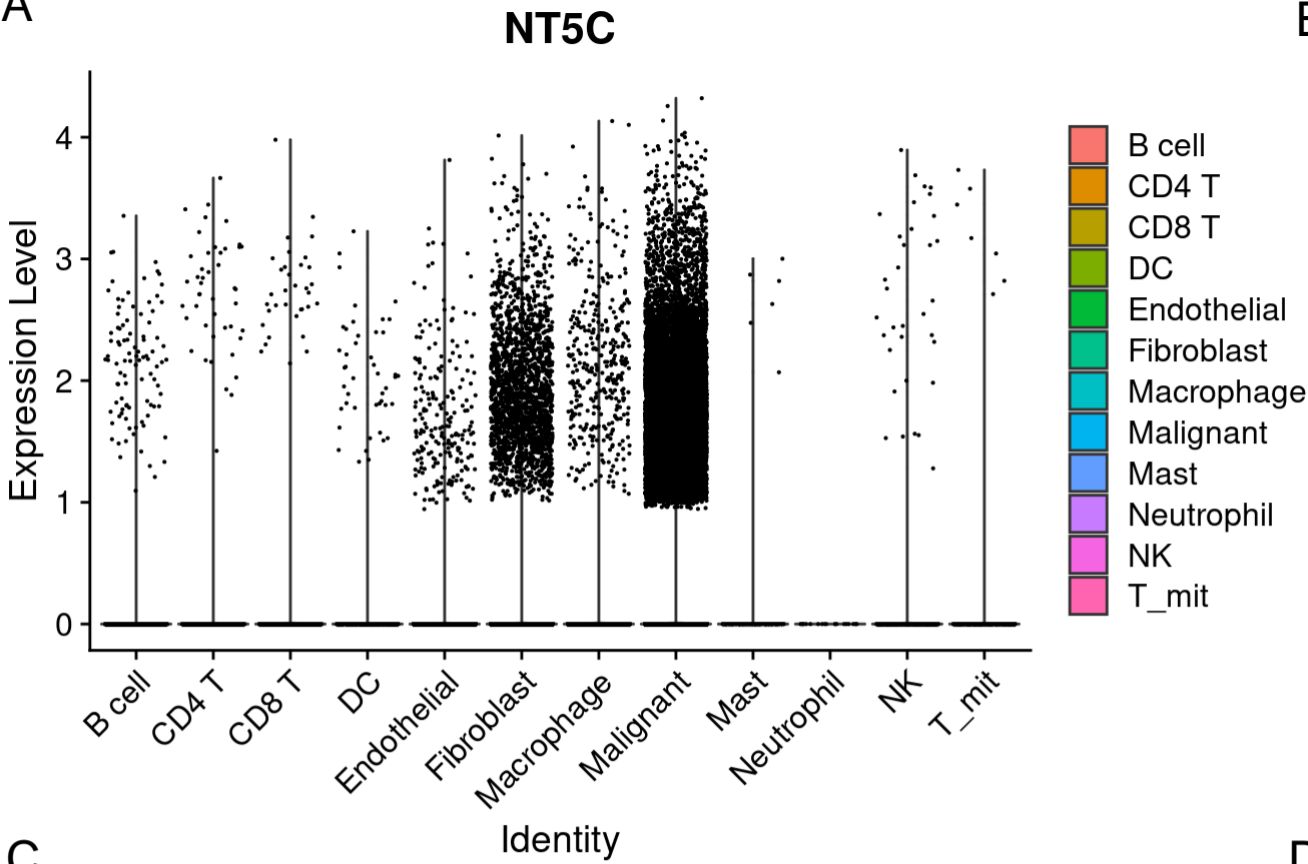

B

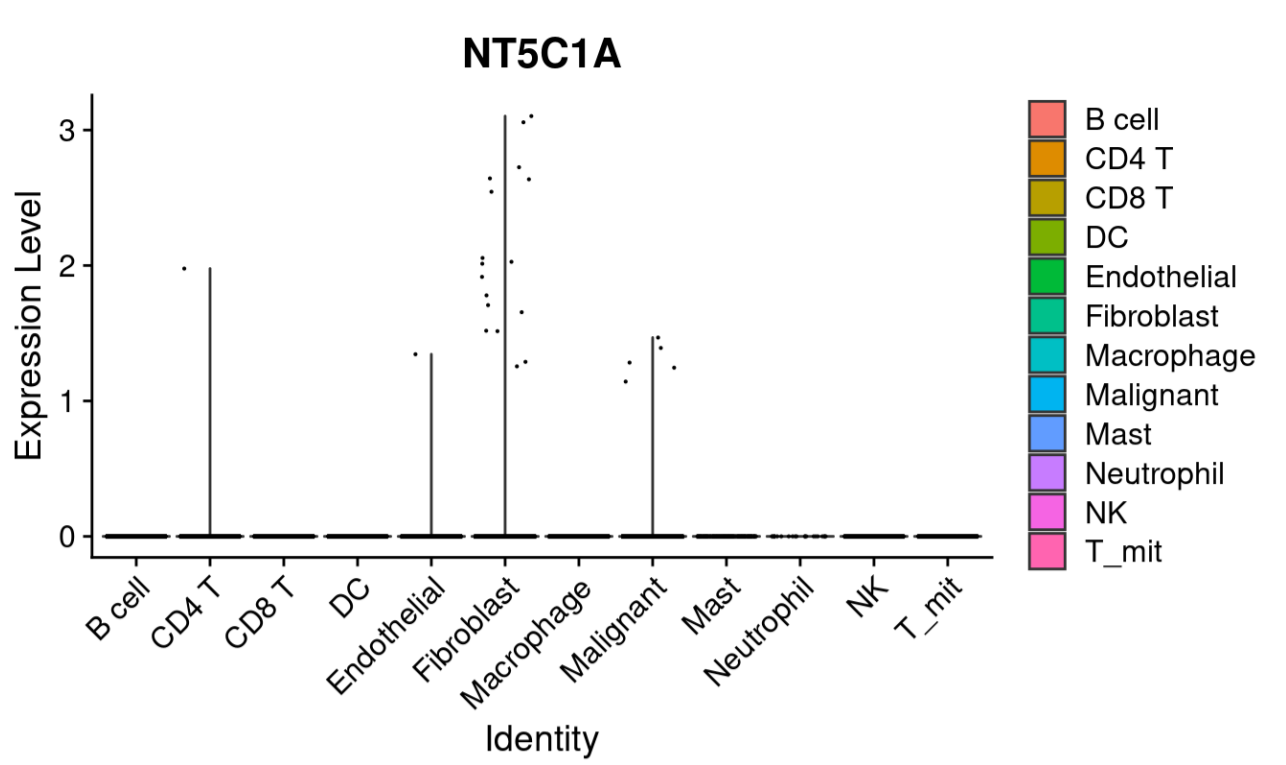

C

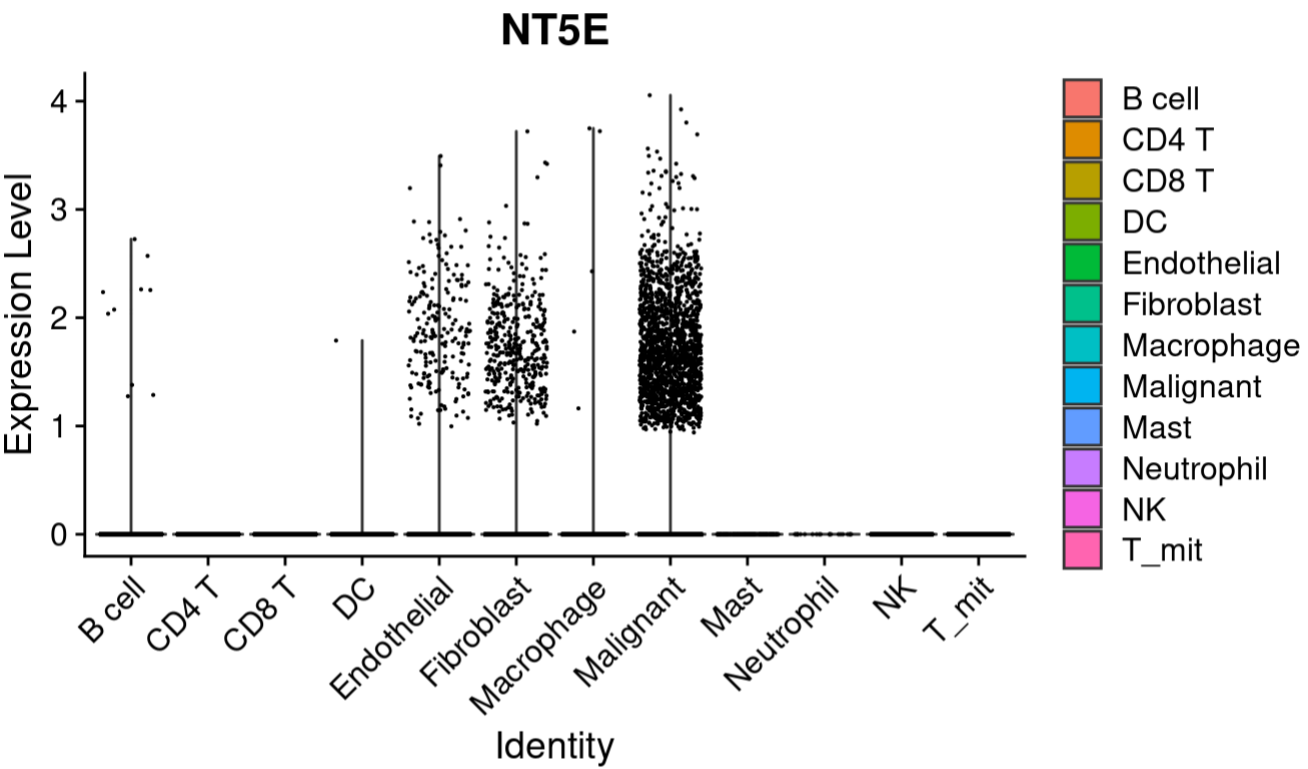

D

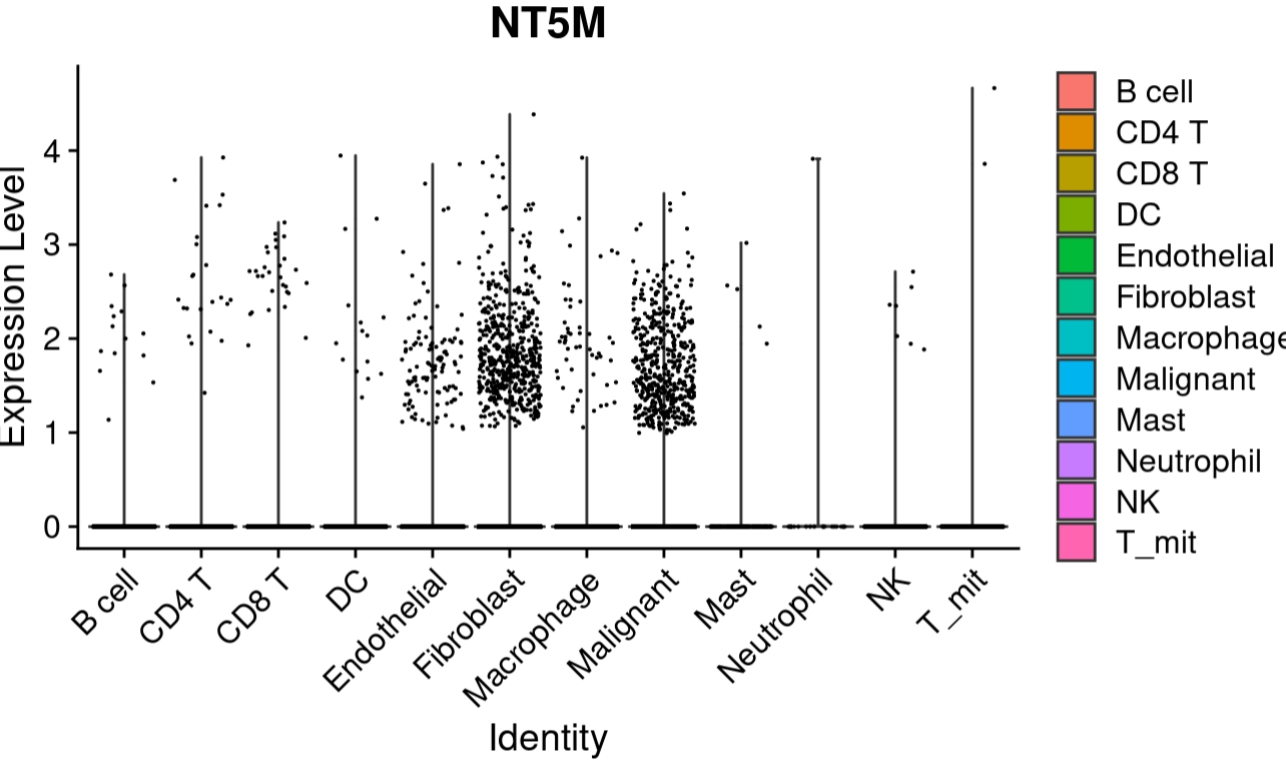

E

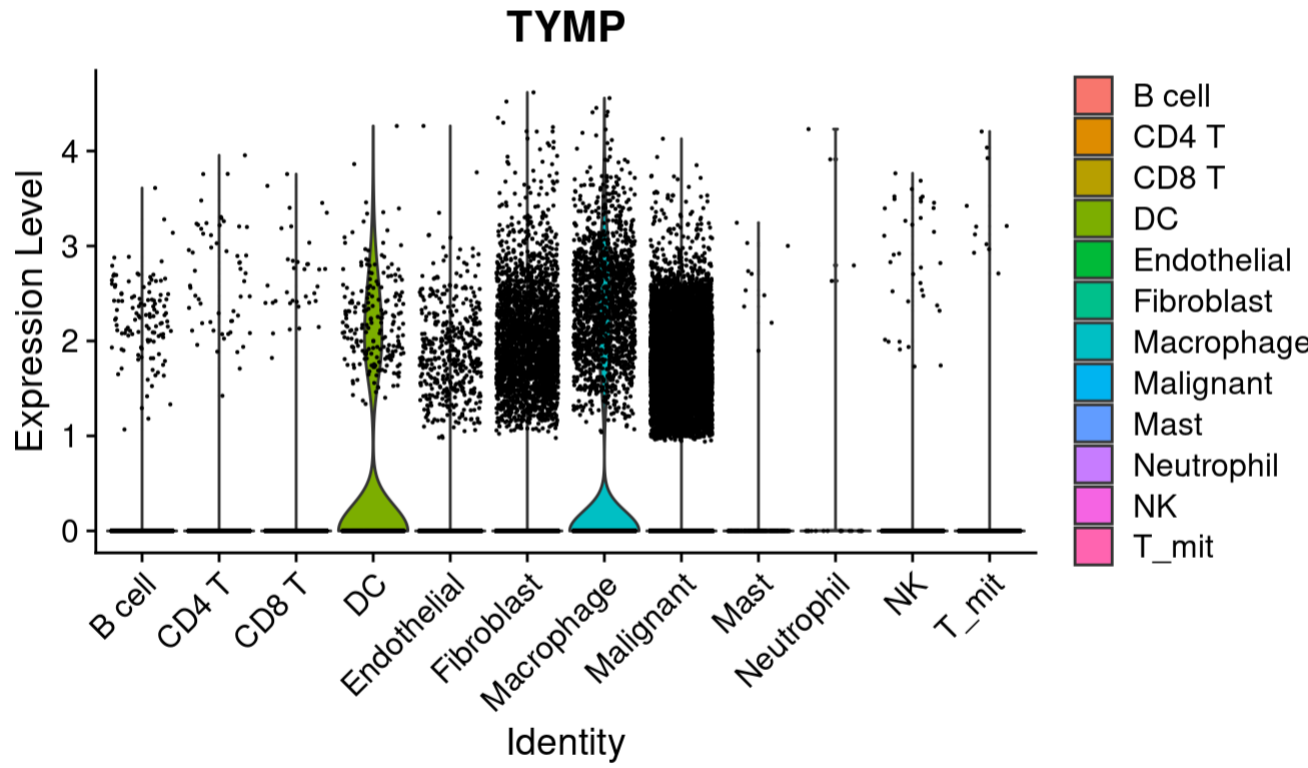

F

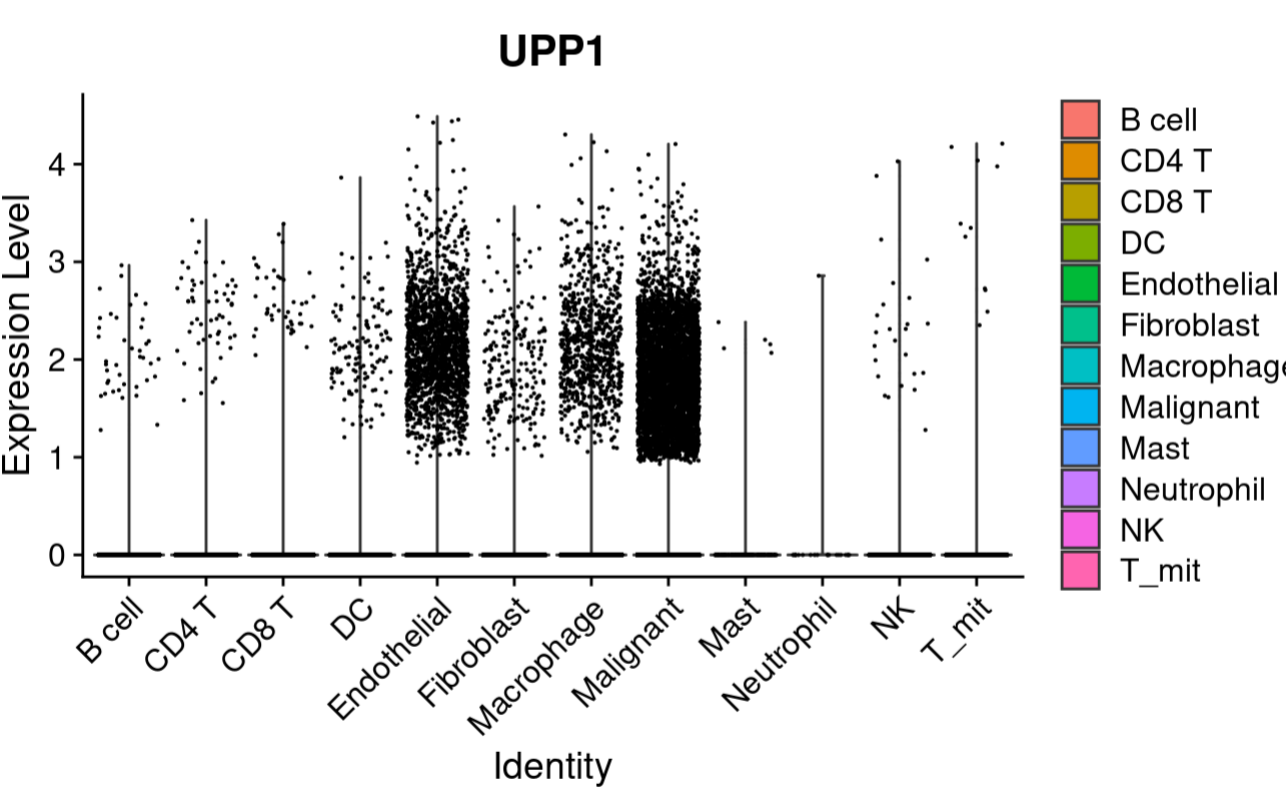

G

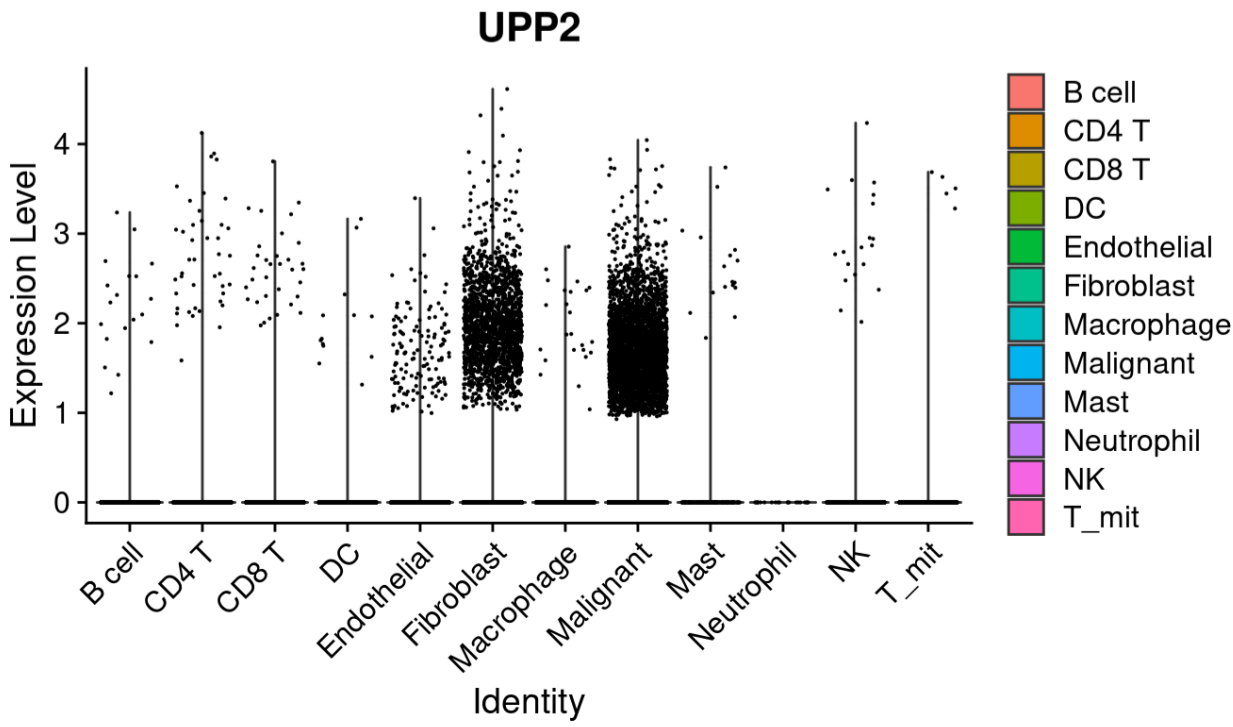

Supplement: Supplementary file 6 [file js9-110-3591-s009.pdf]
